# Associations between the Gut Microbiota, Urinary Metabolites, and Diet in Women during the Third Trimester of Pregnancy

**DOI:** 10.1016/j.cdnut.2022.100025

**Published:** 2022-12-24

**Authors:** Eliot N. Haddad, Nikita H. Nel, Lauren M. Petrick, Jean M. Kerver, Sarah S. Comstock

**Affiliations:** 1 Department of Food Science and Human Nutrition, Michigan State University, East Lansing, MI, USA; 2 Department of Environmental Medicine and Public Health, Icahn School of Medicine at Mount Sinai, New York, NY, USA; 3 Institute for Exposomics Research, Icahn School of Medicine at Mount Sinai, New York, NY, USA; 4 Department of Epidemiology and Biostatistics, College of Human Medicine, Michigan State University, East Lansing, MI, USA

**Keywords:** pregnancy, microbiota, gastrointestinal, stool, urine, bacteria, metabolites, diet, liquid chromatography

## Abstract

**Background:**

Pregnancy causes many metabolic and physiologic changes. However, associations between gut microbiota, dietary intake, and urinary metabolites are poorly characterized in pregnant women.

**Objectives:**

The research objective was to identify dietary and microbial associations with urinary metabolites during pregnancy to elucidate potential biomarkers and microbial targets to improve maternal-fetal health. This is a secondary outcome of the study.

**Methods:**

Pregnant women (*n* = 27) in the Pregnancy EAting and POstpartum Diapers pilot study provided dietary intake information in addition to fecal and urine samples at 36 wk gestation. The gut microbiota was characterized following fecal DNA extraction and 16S rRNA gene sequencing. Urinary metabolites were identified using liquid chromatography high-resolution mass spectrometry.

**Results:**

Urinary glycocholate was consistently and negatively correlated with α-carotene intake. There were 9 significant correlations between microbial taxa and urinary metabolites and 13 significant correlations between microbial taxa and dietary intake. On average, *Bacteroides* were the most abundant taxon in the participants’ gut microbiotas. Notably, the gut microbiotas of some pregnant women were not dominated by this taxon. *Bacteroides*-dominant women consumed more protein, fat, and sodium, and their gut microbiotas had lower alpha diversity than those of nondominant participants.

**Conclusions:**

Several urinary metabolites and microbial taxa were associated with maternal diet and gastrointestinal community composition during the third trimester of pregnancy. Future work should determine the mechanisms underlying the associations identified herein.

## Introduction

Pregnancy is a period of great metabolic and physiologic change to facilitate the growth of fetal and maternal structures, such as the placenta and amniotic fluid [1]. Proteins, fats, carbohydrates, and minerals must be allocated to the development of the fetus, placenta, and amniotic fluid [1]. Steroid hormones must be produced and secreted at high levels to ensure the maintenance of the endometrium and fetus [2]. Metabolism becomes diabetogenic to promote the buildup of glucose stores [1]. Importantly, these changes do not happen in isolation.

Indeed, it has recently been postulated that the human body harbors an equal ratio of bacterial and human cells [3]. For this reason, the human organism can be considered a holobiont [4]. The gut microbiota, which consists of all the microorganisms residing within the gastrointestinal tract, is tied to numerous health outcomes and bodily functions. However, there is still much to learn about how gut bacteria interact with, respond to, and potentially modulate pregnancy’s metabolic and physiologic adaptations. In recent years, the effects of the human gut microbiome on physiologic changes have been observed in relation to pregnancy complications, including gestational diabetes mellitus and hypertension disorders [5, 6]. Understanding these associations is important in its potential to elucidate possible biomarkers and microbial targets which could improve pregnancy outcomes.

The relations between gut microbiota and health are often mediated by diet [[7], [8], [9]]. Once consumed, foods and beverages pass through the gastrointestinal tract, where there are countless opportunities for interaction with commensal microbes. It has been demonstrated that acute (<24 h) dietary change induces shifts in gut microbial composition [10]. Additionally, long-term dietary patterns also associate with distinct community structures of the gut microbiota, known as enterotypes [11]. Classifying enterotypes of pregnant women on distinct diets, such as vegetarianism, has demonstrated unique microbial differences according to health status and gives insight into maternal metabolism and fermentation [12]. Single nutrients or dietary constituents also have important interactions with the gut microbiota. Fiber, for instance, can be metabolized by several gut microorganisms into short-chain fatty acids, which can confer host benefits by reducing inflammation and improving gut barrier function [9, 13].

Metabolomics allows for the characterization of the composition and quantity of the metabolites produced by the human body and microbial symbionts. In turn, this can provide insight into the health status of the human holobiont. Recently, for example, associations between serum levels of imidazole propionate and type 2 diabetes have been linked to the gut microbiome through the induction of impaired glucose metabolism [14]. In a study of pregnant women, proallergic metabolites were associated with microbial diversity and certain gut phyla, demonstrating that there are a wealth of associations that have yet to be discovered in pregnancy [15]. Urinary metabolomics, especially, is an affordable and robust, though underutilized, means of identifying novel hypotheses for the interplay between health, diet, and gut microbiota [[16], [17], [18]]. Unlike circulating metabolites, which are exposed to strong homeostatic forces in human blood, urinary metabolites can reflect more pronounced variations in metabolite concentrations from diet and lifestyle [19]. Indeed, recent work shows that metabolomic analysis of urine can be used to predict individual responses to diet and is, therefore, quite relevant to studies seeking to understand the interplay of the diet with other aspects of human health [20]. However, despite many metabolites being derived from the gut microbiota, current literature fails to establish a baseline urinary metabolic profile for pregnant women and lacks adequate characterization of the relationship between urinary metabolites and microbiota [[21], [22], [23]]. Additional research that employs urinary metabolomic analysis is needed to advance current preliminary results [8, 22].

In this study, our objective was to examine correlations between urinary metabolites, fecal microbes, and dietary intake to better comprehend their interplay with and potential effects on metabolism. We profiled metabolites using liquid chromatography (LC) high-resolution mass spectrometry and performed fecal DNA extractions and 16S rRNA gene sequencing during the third trimester of pregnancy for participants in the Pregnancy EAting and POstpartum Diapers (PEAPOD) study. The characterization of these associations may help elucidate putative biomarkers or metabolic processes for future mechanistic examination and application to improve health during gestation.

## Methods

### Design

This study is a cross-sectional analysis of the associations between pregnancy dietary patterns, the gut microbiome, and the urinary metabolome at 36 wk gestation. This project is part of the PEAPOD pilot study, a prospective dietary intervention trial during pregnancy with follow-up into the postpartum period. PEAPOD was approved by the Michigan State University institutional review board (IRB #16-1515) and has been previously described in depth [24, 25]. In short, PEAPOD was a feasibility study designed to test study implementation procedures and assess the acceptability of a practical nutrition intervention while collecting biospecimens to assess nutrient biomarkers in blood and urine during pregnancy and gut microbiota from maternal and newborn fecal samples collected during pregnancy and postpartum. The PEAPOD dietary intervention and experimental treatment consisted of weekly food delivery of high-fiber foods beginning at 32 wk gestation and continuing until the delivery of the participant’s baby. The analysis presented herein is a secondary outcome of the PEAPOD study. All methods were performed in accordance with federal, state, and local guidelines and regulations, as laid out in section 2 of the Michigan State University Human Research Protection Program manual, as well as the Declaration of Helsinki [25]. Participants gave informed consent, noting that their data, as well as their fecal and urinary samples, could be used in future research studies. Study participants were initially recruited via flyers at their prenatal care visits and incentivized using minimal financial compensation after sample or dietary recall collection. Study participants provided fecal and urinary samples in coded tubes, so samples returned to lab personnel to be analyzed could not be directly identified. Individual results have not been returned to study participants. There were no significant differences in diet, plasma nutrient markers, or gut microbiota between intervention and control groups [24], so all participants were combined into a single study population for this analysis. PEAPOD participants were a convenience sample of pregnant women recruited from a prenatal care clinic in northern Michigan. Participants had to be >18 y and 24–28 wk gestation at the time of enrollment for inclusion in this study.

### Sample collection

Fecal samples were collected remotely in Para-Pak clean vial collection tubes (Meridian Bioscience) with fecal sample collection kits that were provided to the participants. The samples were then mailed to the lab, where they were immediately aliquoted and stored at –80°C upon receipt. The laboratory received fecal samples an average of 3.8 ± 1.9 d (median: 3.5) after collection. Urine samples were collected on-site by medical center staff at 36 wk gestation and were aliquoted and stored at −80°. These samples were then shipped on dry ice to the Icahn School of Medicine at Mount Sinai for untargeted metabolite analysis.

### Data collection

Participant data were obtained through an enrollment questionnaire (*n* = 27) and a 36 wk gestation fecal sample questionnaire (*n* = 26). These interviewer-administered questionnaires collected information on participant race, educational level, smoking history, prepregnancy and current height and weight, age, insurance, and antibiotic use, between other variables. Participant BMI (in kg/m^2^), both prepregnancy and current, was subsequently calculated using the self-reported height and weight measures. In addition, dietary data were collected through the automated self-administered 24-h (ASA24) Dietary Assessment Tool, version 2016 (National Cancer Institute) [26]. The ASA24 computes intake of upwards of 100 dietary constituents based on participant self-report of food consumption over the last 24 h. To harmonize participant results, proximal dietary intake was recorded and only included participants who completed dietary recall within 2 wk of their fecal sample collection date to reflect relative dietary effects on the microbiota best. Habitual results for each participant consisted of a singular participant profile that averaged the dietary constituent levels reported at each recall.

### Microbiome analysis

DNA was extracted from the fecal samples with the MoBio Powersoil DNA Isolation kit (Qiagen). The V4 16S rRNA gene amplification and sequencing followed a previously described protocol [27]. Processing of sequence reads in the mothur software program followed the Illumina MiSeq standard operating procedure [28] using the Michigan State University High-performance Computing Cluster. The phylotype assigned operational taxonomic units using the SILVA reference taxonomy (v128) [29]. Sequences were rarefied to 9000 reads 999 times per sample, averaged, and rounded to the nearest integer. Rarefaction curves confirmed adequate community coverage. Sequence reads and operational taxonomic units were analyzed in R using the vegan package to compute α and β diversity measures [30, 31]. There were 342 identified taxa, of which 17 remained after excluding all taxa with <1% average relative abundance and any taxa that were absent from >50% of the participants.

### Untargeted chemical analysis

Upon receipt by Icahn School of Medicine at Mt. Sinai, urine samples were thawed, vortexed, and diluted down to an average specific gravity of 1.01. For the metabolomics analysis, 20 μL aliquots were combined with 180 μL acetonitrile containing internal standards for protein precipitation, following a previously described protocol [17, 32]. Briefly, the supernatant was transferred to LC vials, where it was analyzed separately using zwitterionic hydrophilic interaction LC (HILIC) in positive ionization mode and reverse phase chromatography in negative mode. This was coupled to a quadrupole-time of flight mass spectrometer with a dual jet stream electrospray ionization source (Agilent Technologies). Nonpolar metabolites were separated at 50°C by first sandwiching 2 μL of the sample between 10 μL of water and then injecting it onto a Zorbax Eclipse Plus C18, Rapid Resolution High-definition column (50 mm × 2.1 mm, 1.8 μm particle size, Agilent Technologies) coupled to a guard column (5 mm × 2 mm, 1.8 μm Agilent Technologies). Polar metabolites were separated at 25°C by injecting 2 μL of sample onto a HILIC SeQuant zwitterionic-HILIC column (100 mm × 2.1 mm, 100 Å, 3.5 μm particle size, Merck). Samples were analyzed in randomized order.

Metabolites were identified based upon database matching using the in-house Personal Chemical Database Library and Profinder software (Agilent Technologies) considering retention time, accurate mass, isotope distribution, and mass spectrometry/mass spectrometry (MS/MS) fragmentation pattern (when available) matching with reference standards analyzed under the same conditions. This provided the highest identification confidence level (level 1 or 2) based on Metabolomics Standards Initiative criteria [33]. The in-house library identified a total of 277 metabolites (positive = 188; negative = 89) in our sample of pregnant women. After removing duplicates and those with >50% missing values between the 26 participants with metabolite data, 206 metabolites remained for statistical analysis.

### Statistical analysis

The metabolite, microbe, and diet data were loaded into R for statistical analysis [30]. A Spearman correlation analysis was performed between 2 sets of data at a time (i.e., diet by metabolite, metabolite by microbes, or microbes by diet) to improve the power for detecting correlations. Any correlations between the same data set (e.g., microbe by microbe correlations) are not presented here. Alpha levels were set to 0.05. To account for the multitude of correlation tests being performed, false discovery rate adjustment was performed using the Benjamini-Hochberg method, with Q < 0.1 being included. A sensitivity analysis was performed for the participants with habitual dietary intake information. To ensure robust results, the observed correlations with proximal dietary intake were verified with the habitual intake information of a subset of *n* = 18 participants who also submitted both proximal and habitual dietary intake information. We completed a sensitivity analysis removing individuals who were potential outliers from the gut microbial taxa dataset. When those individuals were removed from the correlational analysis, correlation coefficients were similar, and the dietary intake of these individuals was similar to that of other participants who submitted proximal dietary data.

Participants were classified into *Bacteroides-dominant* and nondominant groups. Those individuals in which *Bacteroides* had the highest average relative abundance of all taxa within their gut microbiota were considered *Bacteroides* dominant. Alpha and β diversity were calculated using the vegan package in R [31] and were used to characterize participants’ gut microbiomes between *Bacteroides* groups (dominant compared with nondominant). Alpha diversity indices used include Chao1, Shannon, and Inverse Simpson, which provide insight into the richness and overall diversity of the gut microbiome. These indices were compared between groups of participants using a *t* test if normally distributed and a Wilcoxon Mann-Whitney U test if not, with Shapiro-Wilk to confirm normality. This approach was also used to identify dietary and metabolite differences, in addition to differences in continuous participant characteristics, between *Bacteroides* dominance groups. Categorical participant characteristics were compared between groups using a χ^2^ test.

Beta diversity represents microbial differences between groups. It is visualized by plotting the Sorensen and Bray-Curtis dissimilarities, which utilize information on richness and overall diversity, respectively, with principal coordinates analysis (PCoA). Using the adonis function of the vegan R package, permutational multivariate ANOVA was used to test for significant differences in β diversity between *Bacteroides-dominant* groups. Using the betadisper function of the vegan R package, permutational analysis of multivariate dispersion was used to test for differences in group dispersion. The MASS package was used to conduct a negative binomial regression to identify taxa differences between *Bacteroides-dominant* and nondominant groups [34].

## Results

### Study participants shared similar demographics and provided both proximal and habitual dietary recall

**FIGURE 1:**
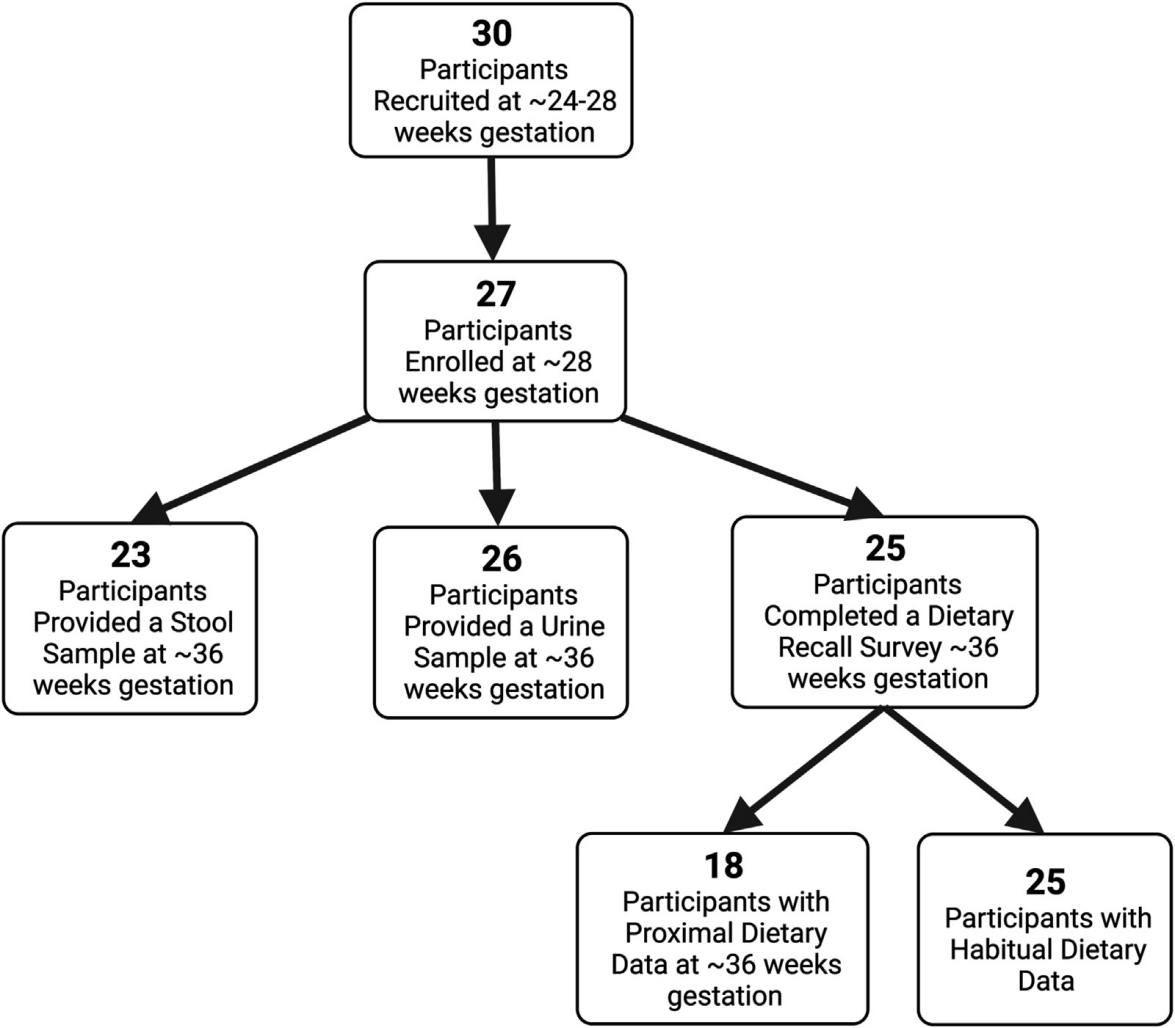
Participant recruitment and enrollment flowchart.

Of the women who originally enrolled in PEAPOD (*n* = 27), 4 did not provide a fecal sample at 36 wk gestation and were therefore excluded from this analysis (Figure 1). All but one participant (*n* = 26) provided a urine sample (Figure 1). Throughout the PEAPOD study period (∼28 wk gestation to ∼6 wk postpartum), all but 2 participants (*n* = 25) completed at least 1 (mean: 4.6, SD: 2.0, median: 5) ASA24 (habitual dietary intake) (Figure 1). Of these, *n* = 18 completed at least 1 (mean: 1.8, SD: 0.5, median: 2) of their recalls within 2 wk of fecal sample collection at 36 wk gestation (proximal dietary intake) (Figure 1). Most participants were White, and nearly half had at least a 4-y college degree. Participants had an average BMI of 27.4 prior to pregnancy, and the average age was ∼30 y (Table 1). Less than 10% (*n* = 2) of women in our study were using either a probiotic or prebiotic, and no participants were taking antibiotics at the time of sample collection.

**TABLE 1:** Participant characteristics for the full study sample, as well as *Bacteroides*-dominant and nondominant groups

Characteristic	Overall (*n* = 23)	*Bacteroides* dominant (*n* = 16)	*Bacteroides* nondominant (*n* = 7)	Test statistic	*P* value
Race (White - non-Hispanic)	19 (82.6)	14 (87.5)	5 (71.4)	5.60^1^	0.06
Race (White - Hispanic)	2 (8.7)	2 (12.5)	0 (0)		0.06
Race (mixed)	2 (8.7)	0 (0)	2 (28.6)		0.06
4+ y of University education	11 (47.8)	9 (56.3)	2 (28.6)	0.59^1^	0.44
Ever smoked	8 (34.8)	5 (31.3)	3 (42.9)	0^1^	0.95
Prepregnancy BMI	27.4 ± 6.9	27.4 ± 7.5	27.2 ± 6.0	54.00^2^	0.92
Prepregnancy BMI (normal)	12 (52.2)	8 (50.0)	4 (57.1)	0.33^1^	0.85
Prepregnancy BMI (overweight)	6 (26.1)	4 (25.0)	2 (28.6)		0.85
Prepregnancy BMI (obese)	5 (21.7)	4 (25.0)	1 (14.3)		0.85
Current BMI	31.2 ± 6.4	31.4 ± 7.1	30.8 ± 5.0	54.50^2^	0.95
BMI category (normal)	3 (13.0)	2 (12.5)	1 (14.3)	0.17^1^	0.92
BMI category (overweight)	8 (34.8)	6 (37.5)	2 (28.6)		0.92
BMI category (obese)	12 (52.2)	8 (50.0)	4 (57.1)		0.92
Age	29.7 ± 4.3	29.1 ± 3.8	31.0 ± 5.4	−0.86^3^	0.41
Medicaid	5 (21.7)	4 (25.0)	1 (14.3)	0^1^	0.98
Antibiotics in the past year (1+ times)	9 (39.1)	8 (50.0)	1 (14.3)	1.32^1^	0.25

BMI, body mass index.

1 Pearson’s χ2 test

2 Mann-Whitney U test

3 *t* test

### Urinary glycocholate is consistently and negatively correlated with α-carotene intake

**FIGURE 2:**
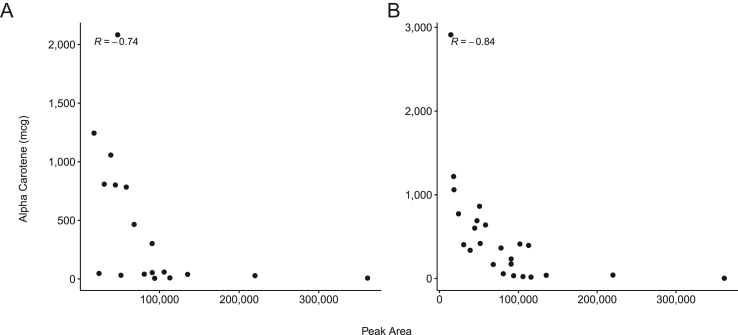
Urinary glycocholate is negatively correlated (Spearman; Q < 0.1) to (A) proximal and (B) habitual α-carotene intake.

Correlation analysis of urinary metabolites with dietary intake revealed numerous significant relations. There were 24 significant correlations between proximal dietary intake and urinary metabolites in pregnant women (Supplemental Table 1). In addition, there were 15 significant and robust correlations between habitual dietary intake and urinary metabolites (Supplemental Table 2). With respect to proximal and habitual diet, only urinary glycocholate and α-carotene were consistently associated (Figure 2).

### Many microbial taxa are significantly associated with urinary metabolites and dietary intake

**FIGURE 3:**
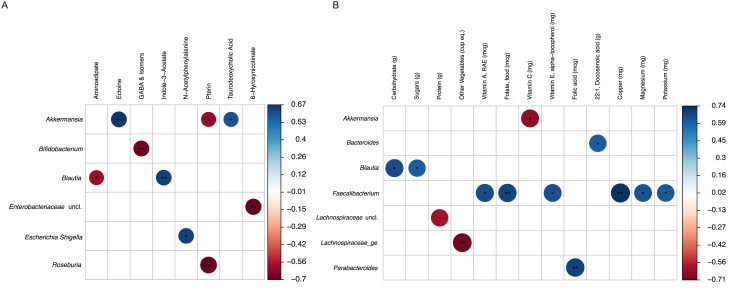
Correlation plots of taxa significantly associated with (A) urinary metabolites and (B) dietary constituents. ∗Q<0.1; ∗∗Q<0.05. GABA, γ-aminobutyric acid.

Numerous taxa of the gut microbiome were associated with urinary metabolites or dietary intake (Figure 3). In addition, there were 9 significant correlations between microbial taxa and urinary metabolites (Supplemental Table 3 and Supplemental Figure 1). There were 13 significant correlations between microbial taxa and proximal dietary intake (Supplemental Table 4 and Supplemental Figure 2). *Blautia* and *Akkermansia* were the 2 genera that were either positively or negatively associated with both urinary metabolites and proximal diet. However, the dietary constituents and urinary metabolites associated with those 2 taxa were not correlated with one another (Supplemental Table 1).

### Participants can be categorized by *Bacteroides* dominance in their gut microbiomes

A PCoA plot of the Bray-Curtis distances of the participants’ gut microbiotas revealed that *Bacteroides* drove most of the variation in β diversity (Supplemental Figure 3). Hence, participants were grouped into *Bacteroides-dominant* and nondominant groups, with dominance being defined as having *Bacteroides* as the single most abundant taxon in one’s gut microbiome. Of the 23 participants, 16 were *Bacteroides* dominant, with the *Bacteroides* percent abundance ranging from 18% to 66%. There were no significant differences in participant characteristics between *Bacteroides-dominant* compared with nondominant groups (Table 1).

### *Bacteroides-*dominant individuals are more likely to consume proteins, fats, and sodium

**FIGURE 4:**
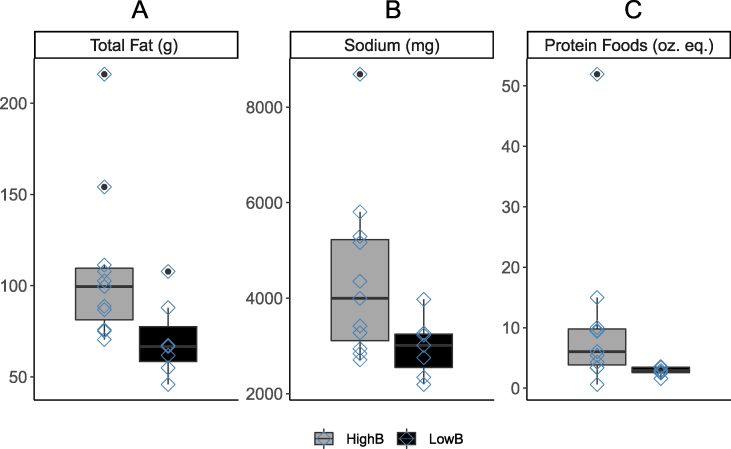
*Bacteroides-dominant* individuals (HighB) consume more total fat, sodium, and proteinaceous foods (*P* < 0.05) than *Bacteroidesnondominant* individuals (LowB). In this study, proteinaceous foods include total meat, poultry, organ meat, cured meat, seafood, eggs, soy, and nuts and seeds, but exclude legumes.

Participant consumption of 8 proximal dietary constituents differed significantly between *Bacteroides-dominant* and nondominant groups (Supplemental Table 6). Notably, proteinaceous foods (excluding legumes), total fat, and sodium were all reported to have been consumed in significantly greater quantities between *Bacteroides-*dominant individuals (Figure 4). In addition, 6 urinary metabolites differed significantly between *Bacteroides* groups (Supplemental Table 5). However, none of the differences in both dietary intake and urinary metabolites remained significant after applying the false discovery rate correction.

### Gut microbial diversity of *Bacteroides*-dominant individuals significantly differs from non–*Bacteroides*-dominant individuals

The non–*Bacteroides-*dominant group had significantly higher α diversity than the *Bacteroides-*dominant group for all indices used (Supplemental Figure 4A, B, and C). Beta diversity between the 2 groups also differed significantly, as shown by the PCoA of the Sorensen (Supplemental Figure 4D) and Bray-Curtis (Supplemental Figure 4E) dissimilarities for the participants. There were 3 taxa, in addition to *Bacteroides*, that significantly differed in abundance between the gut microbiotas of *Bacteroides-dominant* compared with nondominant individuals when observing a negative binomial distribution (Supplemental Figure 4F). The genera *Prevotella 9* was enriched in nondominant individuals. The negative binomial distribution of *Phascolarctobacterium* differed significantly between *Bacteroides-dominant* and nondominant groups, as did the distribution of *Acidaminococcus*. Interestingly, the median relative abundance of *Phascolarctobacterium* was higher in nondominant individuals, though the mean was higher in the *Bacteroides-dominant* group. For *Acidaminococcus,* the median relative abundance was zero for both groups.

## Discussion

The gut microbiota has a complex interplay with host physiology that is dependent on numerous, often interrelated, factors. By examining correlations between gut microbial taxa and diversity with diet and urinary metabolites, we lay the groundwork for developing important insights into the physiology of pregnancy, a period of great metabolic upheaval. In our sample of pregnant women, glycocholate was consistently and negatively correlated with α-carotene intake. There were 9 significant correlations between microbial taxa and urinary metabolites and 13 significant correlations between microbial taxa and dietary intake. On average, *Bacteroides* were the most abundant taxon in the participants’ gut microbiotas. Notably, the gut microbiotas of some pregnant women were not dominated by this taxon. *Bacteroides*-dominant women consumed more protein, fat, and sodium, and their gut microbiotas had lower α diversity than nondominant participants.

Urinary metabolites are typically water-soluble waste products of metabolism. We adopted a database-dependent metabolomics approach because much of the urinary metabolome remains unknown and inadequately characterized [35]. The urine metabolome is reflective of both habitual diet and recent intake [20, [36], [37], [38], [39]]. Our results add to existing correlations in the literature, of which little is understood about carotenoid excretion pathways [40]. Specifically, the robust negative correlation between α-carotene intake and urinary glycocholate was the sole consistent correlation between a metabolite and both habitual and proximal diet. As early as 1964, the role of glycocholate in the uptake of β-carotene in the intestines of animal models has been described [41]. In our study, proximal β-carotene intake was negatively correlated with glycocholate excretion in urine. In addition, habitual intake of red/orange vegetables (excluding tomatoes), which are rich sources of carotenoids, was also negatively correlated to urinary excretion of glycocholate. Together, these results lend further credence to the potential role of glycocholate in carotenoid absorption and metabolism.

There were several correlations between the gut microbiota and proximal diet or urinary metabolites. Proximal, rather than habitual, diet was analyzed for correlations with taxa because it has been demonstrated that recent intake is robustly associated with gut microbial changes [42]. For the urinary metabolome, fecal samples collected proximal to the time of urine sampling provide insight into the specific microbial taxa that may produce, metabolize, or modify different metabolites of interest [16]. Specifically, *Bifidobacterium* was negatively correlated with urinary aminobutyric acid isomers. This is notable because *Bifidobacterium* is a key constituent of the healthy gut microbiome and is a major component of many probiotic formulations [43]. Additionally, the γ-aminobutyric acid (GABA) isomer is a major inhibitory neurotransmitter of the central nervous system. GABA has been identified as a key microbial metabolite because its production depends on specific gastrointestinal bacterial strains. In previous work, certain strains of *Bifidobacteria* and *Bacteroides* have been demonstrated to produce large quantities of GABA [[44], [45], [46]]. Our study suggests that the gut microbiome and GABA isomers are linked in pregnant women as well, though the inverse association merits further examination in this specific population to better inform health care recommendations regarding probiotic use during pregnancy.

*Blautia* and *Akkermansia* were each correlated to diet and urinary metabolites. *Blautia* was negatively correlated with the urinary metabolite aminoadipate and positively correlated with urinary indole 3 acetate. In addition, *Blautia* was positively correlated with total dietary carbohydrates and total dietary sugar in proximal dietary intake. Previously, *Blautia* has been repeatedly demonstrated to be positively associated with type 2 diabetes [47]. Our results suggest that this association may be mediated by sugar/carbohydrate intake, also correlated to *Blautia*. Of note, although aminoadipate has been suggested to be a marker of hyperglycemia-induced oxidative stress and prediabetes [48, 49], which would suggest that it should be positively correlated with other taxa associated with diabetic conditions, it is negatively correlated to *Blautia* in this study.

*Akkermansia* was positively correlated with the urinary metabolites ectoine and taurodeoxycholic acid and negatively correlated with the urinary metabolite pterin. In addition, *Akkermansia* was negatively correlated to vitamin C intake in proximal dietary intake. *Akkermansia* has been shown to be a key player in regulating the intestinal barrier and inflammation, with important health consequences for the host in regard to mitigating diet-induced obesity and its effects on host metabolism [50, 51]. Oral administration of ectoine has also been recently shown to improve gut barrier function in rats through its regulatory effects on inflammation [52]. Considering the correlation reported herein, it is possible that *Akkermansia* and ectoine’s beneficial effects on the intestinal epithelium may be related. Additionally, the inverse association between vitamin C and *Akkermansia* has been previously demonstrated in a pilot study examining the effects of vitamin C supplementation on the gut microbiotas of 14 healthy individuals [53].

Of all the genera comprising the human gut microbiome, *Bacteroides* are the most abundant [54]. This bacterial taxa has been well-established throughout the literature as a predominant gut enterotype, which aligns with how study participants were categorized [55, 56]. Several *Bacteroides* species have been associated with a wide array of both beneficial and deleterious impacts on the host, and it has also been used to classify the gut microbiome into distinct enterotypes based on its relative abundance [56, 57]. Considering this precedent and that most of the variation in β diversity was explained by *Bacteroides* within our study sample, we classified participants based on the dominance of *Bacteroides* in their gut microbiome. The lack of differences in demographic/behavioral characteristics indicates that enterotype classification is independent of such factors, as has been previously reported [57]. However, future studies should consider whether a participant’s enterotype may be impacted by antibiotic use. Although behavioral characteristics were not significantly different within our study sample, *Bacteroides-dominant* participants more commonly reported antibiotic use within the year prior to participation in the study (Table 1). Given that members of the genera *Bacteroides* have been reported to have a high level of resistance to β-lactam antibiotics, antibiotic exposure may be an overlooked determinant for enterotype classification, as previously identified in other study samples [[58], [59], [60]].

We also found that β diversity differed significantly between *Bacteroides-dominant* groups. Indeed, a previous study also observed decreased microbial diversity in individuals with the *Bacteroides* enterotype [61]. In our sample, those who were not dominated by *Bacteroides* were likely to be dominated by *Prevotella* *9*, which is in corroboration past findings that *Prevotella* is another key genus in enterotype classification schemes [57]. Enterotypes are thought to be driven by long-term dietary behavior [11]. The *Bacteroides* enterotype has been associated with greater animal fat/protein consumption, which characterizes the Western pattern diet. The *Prevotella* enterotype, on the other hand, is associated with high-fiber and low animal fat/protein consumption. Prior to false discovery rate correction, *Bacteroides-dominant* participants of this study were more likely to report increased consumption of protein-rich foods (excluding legumes), total fat, and sodium. These dietary constituents are more likely to be found in processed foods typical of the Western diet, which lends further credence to the previously reported dietary associations with the *Bacteroides* enterotype. In contrast, there was no association with the *Bacteroides* enterotype to total carbohydrate intake, despite *Bacteroides* abundance being linked to complex carbohydrate supplementation and metabolism [62, 63]. It is important to note that the digestion of dietary carbohydrates depends on their composition, individual heritability, and health status, which may explain the conflicting findings in this area of study, as well as our own study sample [64, 65]. Considering that high α diversity is associated with numerous health benefits [66] and that the Western pattern diet is associated with numerous negative health outcomes [67], it is worth examining whether public health can be improved through microbiota-based therapies or dietary changes to promote *Prevotella* dominant, rather than *Bacteroides dominant*, enterotypes.

Altogether, our findings reveal important potential insights into the interplay between the gut microbiome, metabolism, and diet in a sample of pregnant women. However, it is prudent to note the limitations of this study when interpreting the results. In this analysis, the purpose was not to compare urinary metabolite correlations between pregnant and nonpregnant women but instead to set a precedent for understanding baseline correlations between urinary metabolites, dietary intake, and the gut microbiome within a pregnant population, which adds novelty to the analysis. As a result, a nonpregnant control group was not included, and this omission restricts our ability to pinpoint specific effects of pregnancy on metabolism and hinders the generalizability of these results to the general population. However, our results are important for establishing unique baseline correlations as a precedent for future studies to explore further. A future direction of this research is to examine differences between pregnant and nonpregnant women to establish specific metabolic and microbial changes as well as associations in pregnancy. Our study population also lacked significant racial diversity and had a narrow range of education levels, mainly those with graduate-level degrees, restricting our external validity. Additionally, participants were recruited as a convenience sample, which may bias the sample towards being more health-conscious than average. Self-reported dietary intake is prone to social-approval bias, and women are twice as likely as men to be prone to this bias [68]. As such, social-desirability bias may have impacted self-reported dietary intake data within our study population. The microbiome was characterized with 16S rRNA gene sequencing, which restricts our ability to describe the full range of the microbiota’s metabolic activity. In the future, similar studies may benefit from utilizing comprehensive metagenomic techniques (i.e., whole genome sequencing) to parse out mechanistic links and underlying metabolic interactions. To obtain a longitudinal understanding of the relations between diet, microbes, and metabolism, it would be helpful to analyze metabolites in multiple urine samples over an extended period during pregnancy and into the postpartum phase. This will allow for a greater understanding of changes that occur from conception into the perinatal period. More sensitive dietary measures would also improve future studies with similar aims. Namely, a food frequency questionnaire or dietary history could be used to identify habitual dietary patterns, and repeated 24-h recalls corresponding to fecal, and urine sampling would provide more exact proximal intake information. Finally, fecal samples were exposed to ambient temperatures during shipment from participants to the laboratory. Nonetheless, validity is maintained due to the methodological consistency, which results in similar microbial changes across all samples and does not prevent our ability to accurately characterize meaningful differences in diversity or composition [69, 70].

In conclusion, multiple correlations between the gut microbiome, urinary metabolites, and dietary intake during pregnancy exist. These results highlight putative microbial-host metabolic interactions and provide grounds for hypothesis generation for mechanistic exploration in future investigations. Furthermore, we demonstrate that, during pregnancy, *Bacteroides* dominance is associated with the consumption of archetypal constituents of the Western pattern diet and that it is also associated with reduced microbial diversity compared with non-*Bacteroides* dominance. These findings are important in their ability to direct future work with a specific focus on tailoring a diet to enhance the gut microbial composition and holobiont metabolism to improve health during pregnancy.

## Acknowledgement

The authors’ responsibilities were as follows: SSC and JMK conceptualized the research, curated the data, determined which methodologies to use, provided resources and supervision, and contributed to the writing, including review and editing. LMP contributed through data curation, formal analysis, methodology, writing–review, and editing. ENH and NHN contributed through formal statistical analysis, visualization, and writing. SSC has primary responsibility for the final content. All authors read and approved the final manuscript.

## Funding

Supported in part through computational resources and services provided by the Institute for Cyber-Enabled Research at Michigan State University and the National Institutes of Health National Institute of Environmental Health Sciences under award number U2CES030859.

## Author disclosures

SSC and JMK were financially supported by start-up funds from Michigan State University. JMK was supported in part by a CHEAR Pilot & Feasibility Program: Project 2018-PF05. ENH was supported by a National Science Foundation BEACON (Bio/computational Evolution in Action CONsortium) Summer Luminary fellowship. NHN was supported by the Michigan State University Honors College Professorial Assistantship program. None of the authors have any conflicts of interest to report.

## Data Availability

Data described in the manuscript, code book, and analytic code will not be made available due to the small sample size from a geographically limited location but are available from the corresponding author at reasonable request.
