# Supplementary material for: Associations between the Gut Microbiota, Urinary Metabolites, and Diet in Women during the Third Trimester of Pregnancy
Source: Curr Dev Nutr. 2022 Dec 24;7(4):100025. doi: 10.1016/j.cdnut.2022.100025 (PMC10257213; doi:10.1016/j.cdnut.2022.100025)

**Supplemental Table 1.** Metabolite correlations with proximal diet.


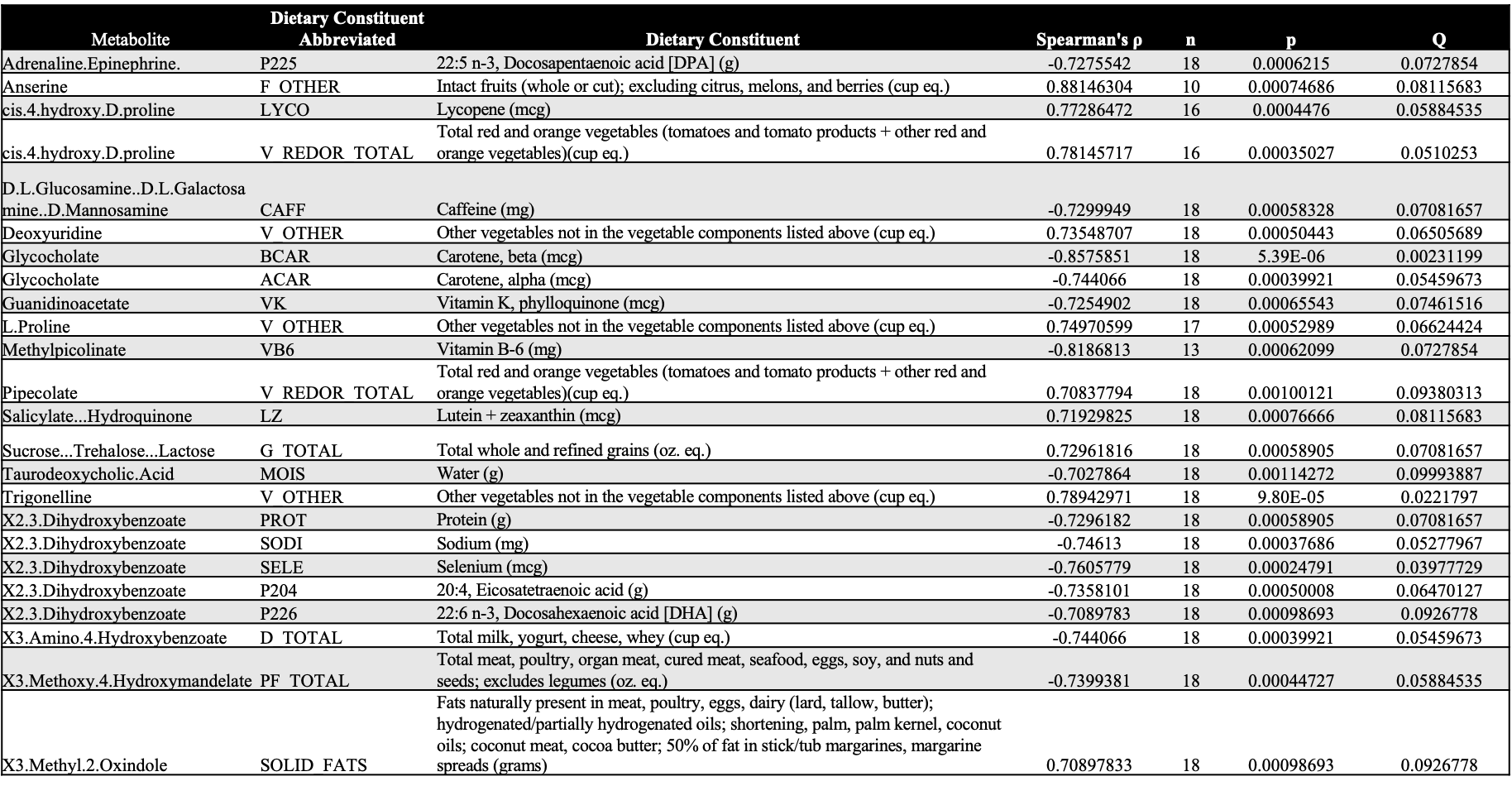


**Supplemental Table 2.** Metabolite correlations with habitual diet.


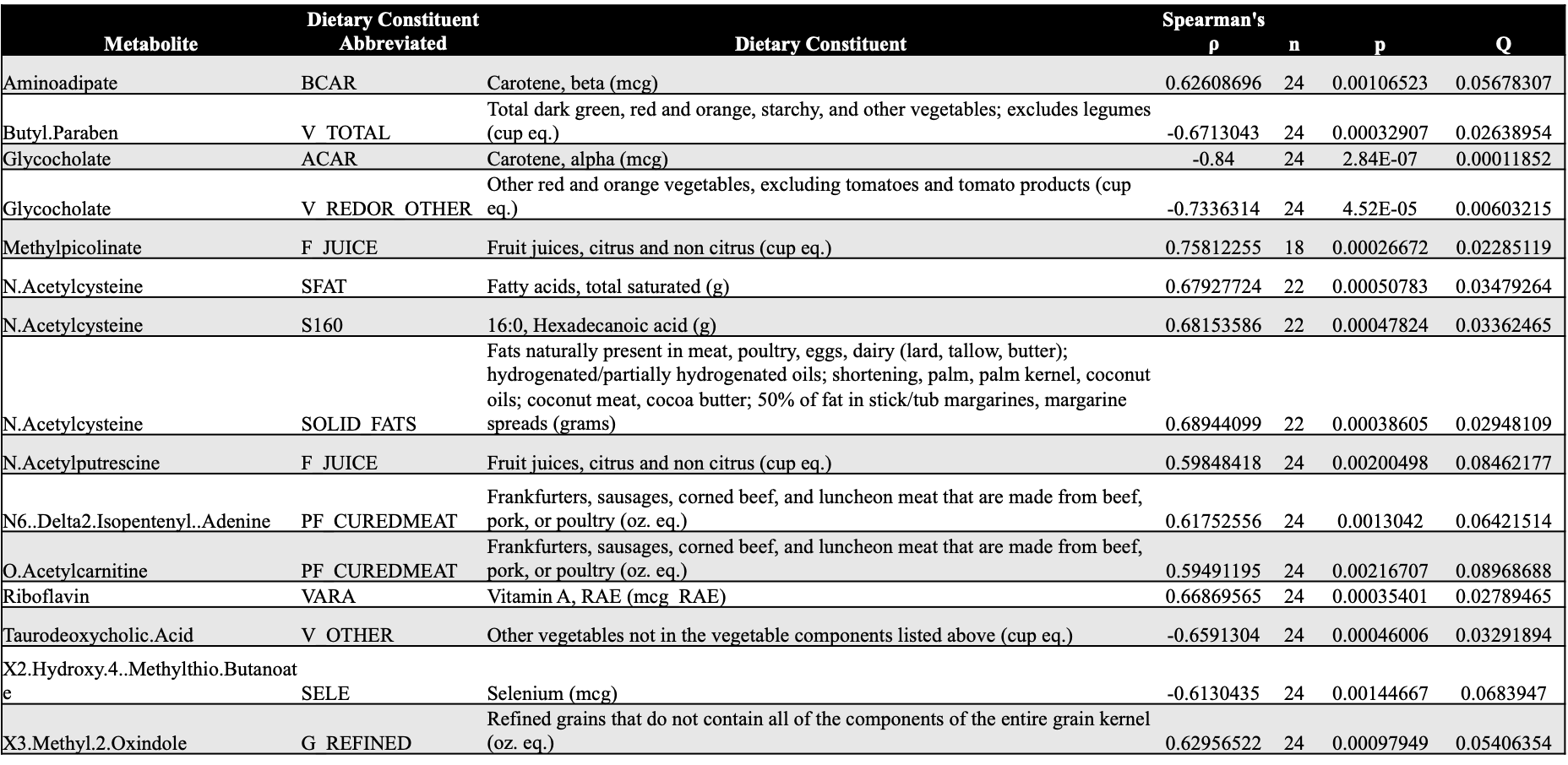


**Supplemental Table 3.** Taxa correlations with metabolites.


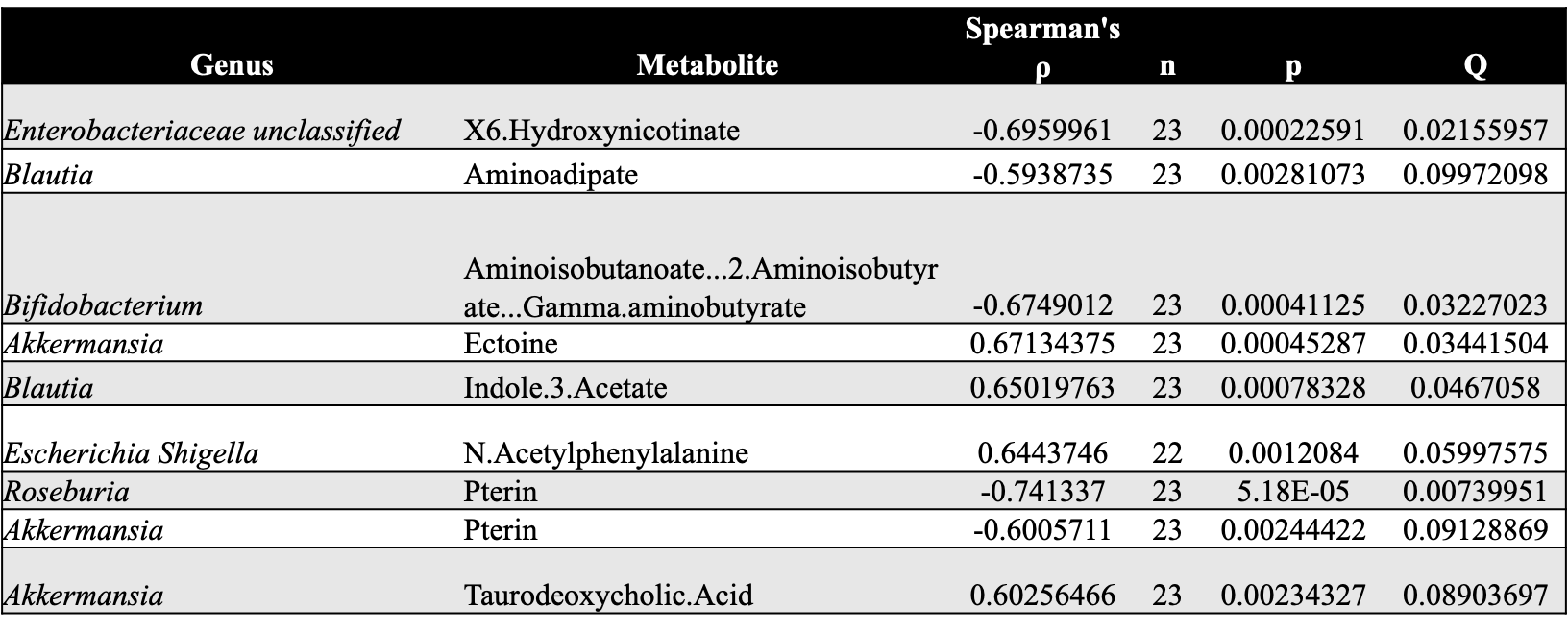


**Supplemental Table 4.** Taxa correlations with proximal diet.


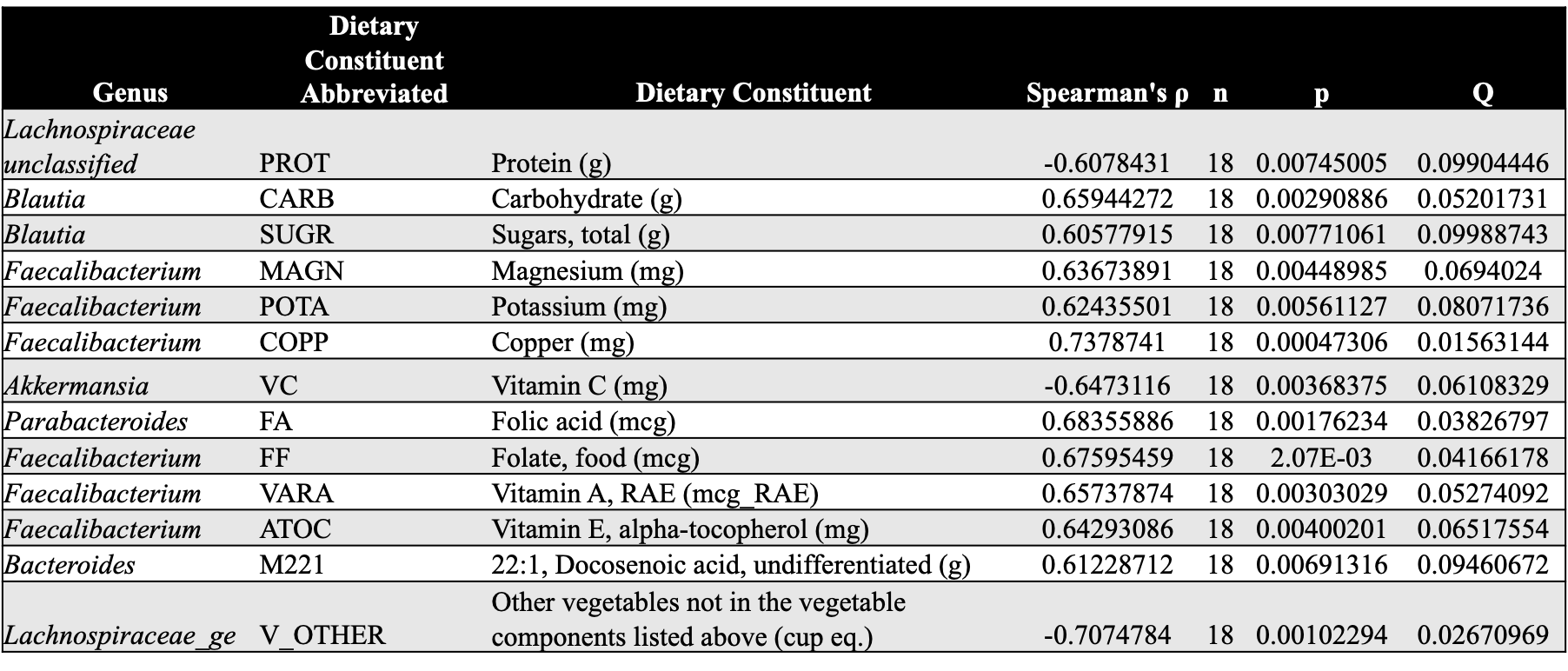


**Supplemental Table 5.** Metabolite differences in *Bacteroides* dominant vs. non-dominant individuals.


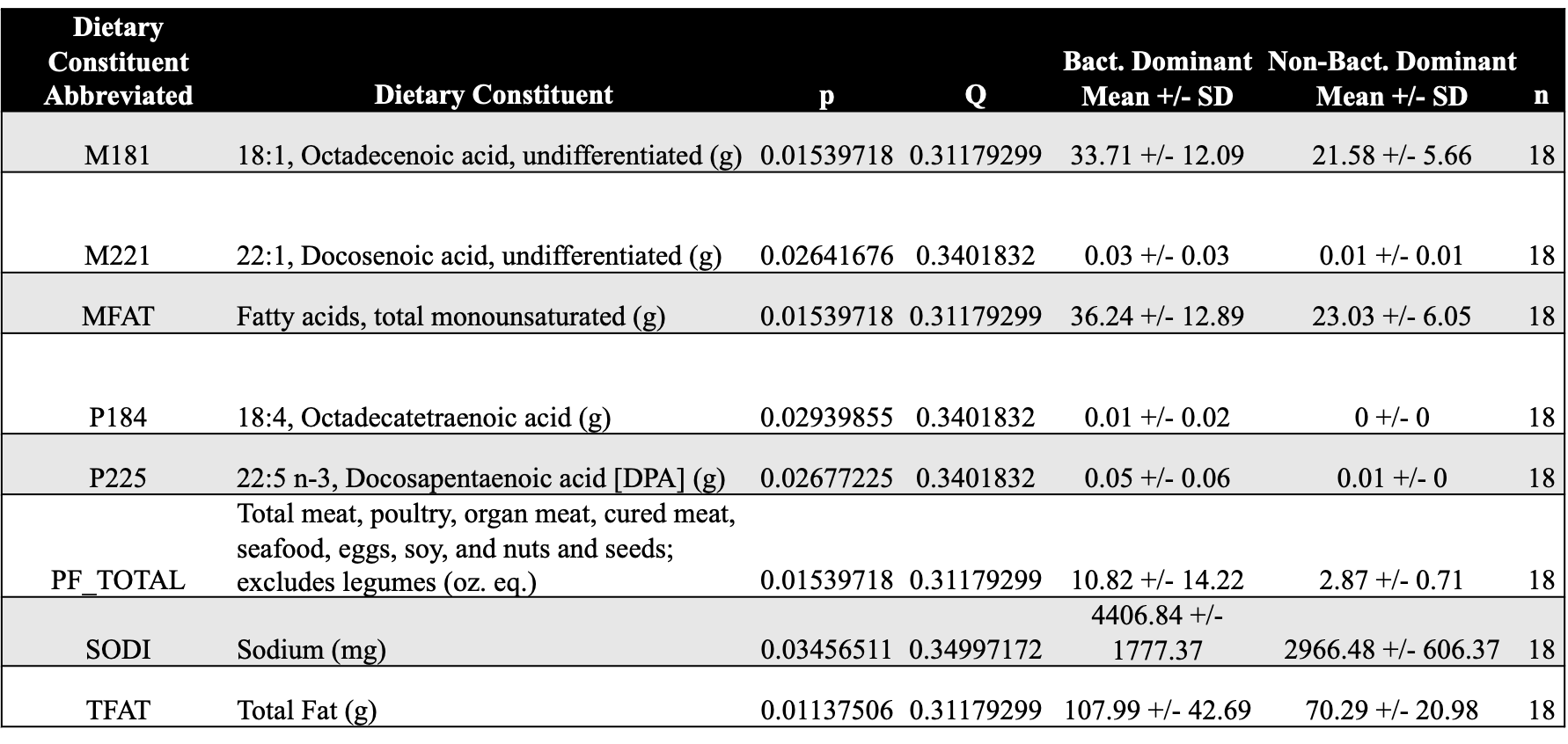


**Supplemental Table 6.** Proximal diet differences in *Bacteroides* dominant vs. non-dominant individuals.

**
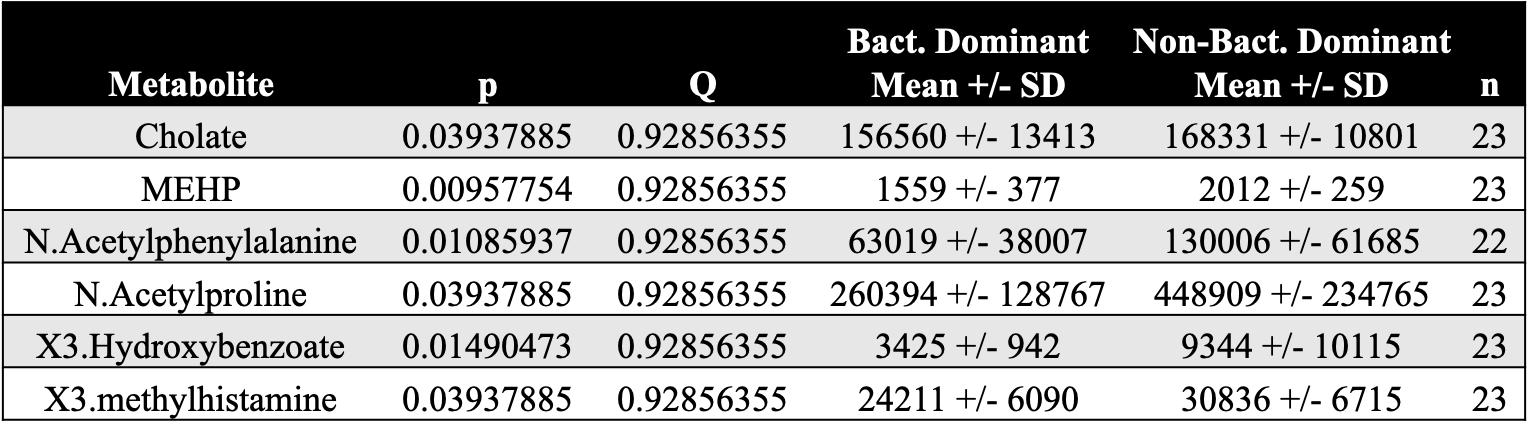
**

**Supplemental Figure 1.** Scatter plots of taxa significantly associated with urinary metabolites (Q<0.1).

**Supplemental Figure 2.** Scatter plots of taxa significantly associated with proximal dietary constituents (Q<0.1).

**Supplemental Figure 3.** *Bacteroides* drives variation in beta diversity.

**Supplemental Figure 4.** There were numerous gut microbial differences between *Bacteroides* dominant (HighB) and *Bacteroides* non-dominant (LowB) individuals. Alpha diversity was significantly lower among *Bacteroides* dominant than *Bacteroides* non-dominant individuals, shown by a) Chao1 (HighB: 89.2 ± 22.6; LowB: 120.6 ± 24.1; p=0.01), b) Shannon (HighB: 2.5 ± 0.5; LowB: 3.0 ± 0.3; p=0.01), and c) Inverse Simpson (HighB: 6.5 ± 3.3; LowB: 11.2 ± 3.8; p=0.02) indices. Beta diversity shown by PCoA of d) Sorensen (p=0.01) and e) Bray-Curtis (p<0.001) distances significantly differed between *Bacteroides* dominant individuals and non-*Bacteroides* dominant individuals. Ellipses depict the standard deviation of points from the centroid of fecal bacterial communities of samples with high versus low *Bacteroides.* f) Four taxa significantly differed between *Bacteroides* dominant and *Bacteroides* non-dominant individuals (Q<0.05).


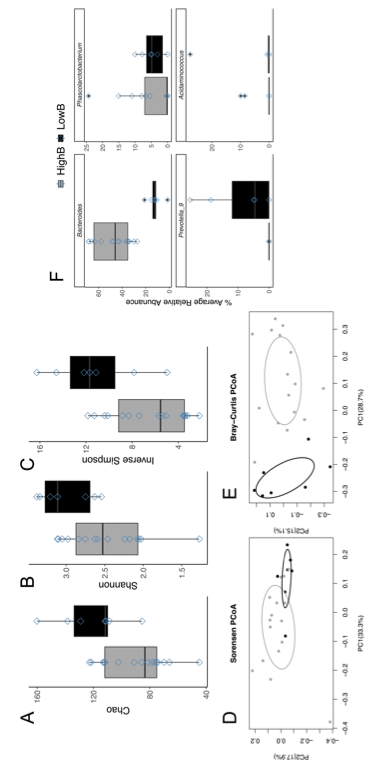

Supplement: Multimedia component 1 [file mmc1.docx]
